# When cold-bloods heat up: meta-analytical evidence that climatic variability mediates behavioural fever in amphibians and reptiles

**DOI:** 10.1098/rspb.2026.1712

**Published:** 2026-11-04

**Authors:** Danilo Giacometti, Leonardo M. Servino, Laura C. Cabanzo-Olarte, Kênia C. Bícego, Carlos Navas

**Affiliations:** ^1^ Departamento de Fisiologia, Universidade de São Paulo, São Paulo, 05508-900, Brazil; ^2^ Departamento de Ecologia, Universidade de São Paulo, São Paulo, 05508-900, Brazil; ^3^ Department of Animal Morphology and Physiology, Universidade Estadual Paulista Julio de Mesquita Filho, Campus de Jaboticabal, Jaboticabal 14884-900, Brazil

**Keywords:** amphibia, body temperature, climate change, disease, pathogens, reptilia

## Abstract

Fever is an adaptive defence response that enhances immune performance by elevating body temperature above normal values. In ectotherms, fever is expressed behaviourally through the selection of warmer microhabitats following infection, yet its magnitude and determinants vary widely across species and environments. We performed a phylogenetically informed meta-analysis to test whether behavioural fever expression is shaped by climatic thermal variability, pathogen identity and taxonomy in amphibians and reptiles. We predicted that species from thermally variable environments would exhibit stronger behavioural fever than those from stable environments and reptiles would exhibit stronger fever responses than amphibians owing to lower hydrothermal constraints. Across 47 studies encompassing 101 effect sizes, behavioural fever emerged as a widespread but context-dependent response. Behavioural fever was strongest in species from thermally variable habitats, suggesting that thermal opportunity and behavioural plasticity mediate the expression of fever. Contrary to our prediction, amphibians exhibited stronger fever responses than reptiles, possibly reflecting differences in thermoregulatory biology, environmental opportunity or methodological artefacts. Bacterial infections elicited larger body temperature increases than fungal or viral challenges. Our findings demonstrate that temperature is not a background condition for host–pathogen interactions, but an active and environmentally contingent component of immune defence in ectothermic tetrapods.

## 1. Introduction

Pathogens represent a pervasive selective pressure in natural systems, shaping host physiology and behaviour, population dynamics and patterns of biodiversity [1]. Exposure to pathogens like bacteria, fungi and viruses is ubiquitous and unavoidable in wild populations [2]. Consequently, hosts have evolved a diverse array of defences to mitigate the costs and consequences of infections. Host defences against pathogens range from molecular and cellular immune responses to behavioural strategies that reduce exposure, limit pathogen growth or enhance immune efficiency [3,4]. Importantly, the expression and effectiveness of these defences depend on factors like ecological context, energetic constraints and the behavioural options available to the host [5–7]. Therefore, understanding host responses to pathogens requires an integrative framework that links immunology, physiology, behaviour and the physical environment in which infection occurs.

Fever is a widespread and biologically important defence response. In vertebrates, fever appears as a pathophysiological reaction to contact with infectious or inflammatory agents (i.e. ‘exogenous pyrogens’) in which core body temperature is regulated above its typical range [8]. The adaptive value of fever lies in its capacity to enhance immune performance and inhibit pathogen replication, making it an effective response across host–pathogen systems [9]. Febrile responses, however, can be achieved through different means that include both autonomic (involuntary) and behavioural responses that vary among taxa [10]. In mammals and birds, fever is generated endogenously through a coordinated process that involves an immune- and endocrine-induced shift in hypothalamic set-points, which is effected through thermogenic and heat-retention mechanisms, such as shivering and non-shivering thermogenesis and/or peripheral vasoconstriction [10–12]. By contrast, amphibians and reptiles produce limited endogenous heat and retain body heat less effectively than endotherms [13]. As a result, fever in amphibians and reptiles is expressed behaviourally through the selection of warmer microhabitats after pathogen exposure [14,15]. Thus, behavioural fever externalizes a physiological process into behaviour, coupling immune activation, movement decisions and habitat use [7,16]. Behavioural fever has been documented across several taxa and is increasingly recognized as a key component of amphibian and reptile immune defence [10,15].

At the physiological level, fever emerges from a cascade of interacting processes that are generally conserved in vertebrates [16]. Exogenous pyrogens are detected by pattern recognition receptors in immune cells, triggering the release of pro-inflammatory cytokines (e.g. interleukin-1ß and tumour necrosis factor), which subsequently stimulate prostaglandin (e.g. prostaglandin E2) production. These mediators alter the neural processing of thermal information and increase thermoregulatory drive, thereby inducing fever [4,17]. In amphibians and reptiles, this cascade does not result in an increase in metabolic heat production; instead, it culminates in shifting locomotory patterns and biases habitat selection toward warmer environments (i.e. positive thermotaxis), ultimately resulting in a regulated increase of thermoregulatory set-points [18–20]. Thus, the expression of behavioural fever depends not only on immune signalling, but also on sensory integration, locomotor capacity and the structure of the surrounding thermal landscape [21–24]. Behavioural fever should therefore be viewed as an emergent property of physiological plasticity interacting with environmental thermal conditions, rather than as a fixed trait expressed uniformly across species or contexts. Importantly, behavioural fever is typically inferred relative to temperatures selected under non-infected conditions (i.e. a reference value considered normal or the ‘baseline thermal state’), such that its interpretation depends on whether experimentally derived thermal preferences represent stable thermoregulatory tendencies or context-dependent responses to laboratory conditions, raising the question of how comparable behavioural fever responses are across taxa [10,25].

Environmental thermal variability is expected to play a central role in shaping behavioural fever in amphibians and reptiles. The climate variability hypothesis (CVH) posits that organisms inhabiting thermally variable environments should evolve broader thermal tolerances and greater physiological and behavioural plasticity than organisms from more thermally stable climates [26,27]. Comparative evidence supports this prediction across multiple ectothermic taxa, with species from more variable environments typically experiencing and tolerating greater variation in thermal biology traits [28–30]. Although the CVH has rarely been examined in the context of behavioural fever [but see 31], it predicts that species exposed to pronounced temperature fluctuations should be better equipped to exploit the thermal landscape as a regulatory tool during infection. For such species, fever-inducing temperatures may fall within the range routinely experienced during activity, potentially reducing the physiological costs of maintaining a febrile state [21,32,33]. Conversely, species from thermally stable environments may incur greater costs when reaching or maintaining fever-inducing temperatures [34], thereby constraining the magnitude of behavioural fever they can express. Under such constraints, alternative sickness behaviours (e.g. behavioural anapyrexia, fasting or reduced activity) may represent potential adaptive responses to infection, highlighting that the absence of behavioural fever should not necessarily be interpreted as an absence of thermoregulatory engagement [25].

Critically, the CVH should not be treated as an inherently latitudinal hypothesis, because its corollaries depend strongly on the scale at which thermal variability is quantified. Contrary to suggestions from preliminary studies, thermal heterogeneity does not increase monotonically from the tropics to higher latitudes, nor is it adequately captured by latitude or longitude alone [35,36]. Instead, thermal variability depends on spatial (e.g. microhabitat versus regional climate) and temporal scales (e.g. diurnal versus seasonal shifts), as well as landscape features (e.g. elevation and canopy cover) [34,37]. These factors are relevant for ectotherms that rely on external sources of heat to behaviourally regulate body temperature during infection. Testing the applicability of the CVH to behavioural fever therefore requires climatic metrics that capture biologically relevant thermal environments rather than coarse geographic proxies [38,39].

Amphibians and reptiles provide an informative comparative system to test the extent to which the CVH predicts the expression of behavioural fever. These groups differ markedly in integumentary permeability, water balance and thermoregulatory patterns [40,41], all of which may influence the expression of behavioural fever. Amphibians possess highly permeable skin and generally show high evaporative water loss, thus being assumed to face strong hydrothermal trade-offs that should limit sustained warming under natural conditions, although the extent to which these constraints are expressed under experimentally permissive settings remains unclear [42–44]. In contrast, reptiles generally possess less permeable integuments and maintain higher body temperatures, leading to the expectation of stronger fever responses [40,45]. Nonetheless, substantial variation exists within and across both clades [25], suggesting that environmental context and evolutionary history may be more informative predictors than taxonomy alone. Pathogen identity adds further complexity, as different pathogens vary in thermal sensitivity and immune activation pathways, potentially influencing both the magnitude and timing of fever responses [4]. Consequently, understanding behavioural fever requires integrating host physiology, pathogen biology and thermal opportunity across ecological contexts.

Despite decades of experimental work, no quantitative synthesis has evaluated whether climatic thermal variability predicts the expression of behavioural fever across ectothermic tetrapods. At the same time, behavioural fever studies differ markedly in experimental design, pathogen exposure and the conditions used to quantify thermal responses, raising important questions about the comparability of fever expression across taxa and ecological contexts. Here, we present a phylogenetically informed meta-analysis of behavioural fever in amphibians and reptiles to test whether fever expression is better explained by climatic thermal variability and pathogen identity than by taxonomic differences alone and to evaluate how environmental context mediates temperature-based defence mechanisms in natural systems. We predicted that species from more thermally variable habitats would express stronger behavioural fever than those from more thermally stable habitats and that this effect would be mediated by both taxonomy (greater effect in reptiles than amphibians) and pathogen identity after controlling for body size effects and shared ancestry. Our study gives insight into factors that may affect behavioural fever in tetrapod ectotherms, and highlights that defence mechanisms cannot be understood in isolation from the environments in which they are expressed.

## 2. Material and methods

### (a) Literature search and inclusion criteria

In August 2025, we conducted a systematic literature search on the *Scopus* and *Web of Science Core Collection* databases following the Preferred Reporting Items for Systematic Reviews and Meta-Analyses statement [46]. On both databases, we used search strings consisting of the following keywords: (‘amphibia*’ or ‘reptile*’ or ‘frog*’ or ‘toad*’ or ‘anura’ or ‘salamander’ or ‘caudat*’ or ‘caecilian*’ or ‘gymnophiona’ or ‘lizard*’ or ‘snake*’ or ‘squamat*’ or ‘turtle*’ or ‘testudine*’ or ‘tortoise*’ or ‘alligator*’ or ‘crocodil*’), (‘infection*’ or ‘disease’ or ‘pathogen*’ or ‘bacteria*’ or ‘fung*’ or ‘vir*’), (‘body temperature’ or ‘Tpref’ or ‘Tsel’ or ‘Tset’), (‘fever’ or ‘behavioural fever’ or ‘behavioral fever’) and (‘thermoregulat*’ or ‘therm*’). We retrieved a total of 791 documents (supplementary material, figure S1) and, after removing 127 duplicates, we assessed the eligibility of the remaining 664 studies by reading their titles and abstracts. As inclusion criteria, we considered studies that: (i) used amphibians or reptiles as study systems, (ii) evaluated animals that were either experimentally or naturally exposed to an immune challenge or pathogen, (iii) stated the type of immune challenge or pathogen (e.g. bacteria or virus), (iv) stated whether animals were raised in captivity or sourced from nature, (v) compared and reported descriptive statistics on the body temperatures of healthy and infected individuals within the same study, and (vi) provided information on body mass. We extracted body temperature means, standard deviations and sample sizes for control and treated/infected groups to calculate standardized mean differences (Hedges’ *g*) and their sampling variances (V*_g_*) [47,48]. We also collated information on species origin, body mass, infection type and geographic location, updating species nomenclature when necessary [49,50]. Further details on data extraction, effect size calculation and data collation are provided in the Supplementary Material.

### (b) Meta-analysis, bias estimates and sensitivity analyses

We used the standardized mean difference (Hedges’ *g*) and its associated sampling variance (*V_g_*) as a measure of effect size (supplementary methods). We performed all analyses using R (version 4.5.1) in RStudio (version 2025.09.2+418) [51] and created our figures with the phytools [52], orchaRd [53], ggplot2 [54] and ggpubr [55] packages. To evaluate study- and experimental-level effects on the expression of behavioural fever, we fitted multi-level meta-analytical models with the ‘rma.mv’ function from the metafor package [56]. We considered *g* as the response variable and *V_g_* as the sampling variance in the meta-analytical models. We included the following random terms in all models: study authorship, origin of study animals, the species’ identity (ID), the phylogenetic relatedness of the species in our dataset and an observation-level random effect (i.e. residual variance). To account for phylogenetic effects, we built a variance-covariance matrix based on recent amphibian and reptile phylogenetic trees [57,58]. We pruned, combined and dated these phylogenies using the ape [59] and geiger [60] packages.

To determine the overall effect size for behavioural fever across amphibians and reptiles, we fitted an intercept-only model. To understand how taxonomy and methodological attributes influenced the expression of fever, we fitted a model that considered taxonomical class (amphibians versus reptiles), origin (nature versus captivity) and infection type (bacterial, fungal, pyrogen, viral or other) as moderator variables. We quantified heterogeneity (*I*^2^) estimates and their associated 95% confidence intervals (CIs) with the ‘i2_ml’ function from the orchaRd package [53], which allowed us to partition the total heterogeneity (*I*^2^_total_) across each random factor in a given model. Thus, we estimated the heterogeneity associated with the identity of the studies (*I*^2^_study_), the origin of animals (*I*^2^_origin_), the species under study (*I*^2^_species_), the phylogenetic relatedness (*I*^2^_phylogeny_) and the residual variance (*I*^2^_residual_). The sum of these *I*^2^ values amounts to *I*^2^_total_, which ranges from 0 to 100% [61]. Generally, 25, 50 and 75% are considered, respectively, as the thresholds for low, moderate and high heterogeneity. Low *I*^2^ values indicate that the observed heterogeneity is the outcome of sampling error, while high *I*^2^ values indicate that heterogeneity is not random [61]. We also estimated the phylogenetic signal attributed to our models with the phylogenetic heritability index (*H*^2^) [62]. *H*^2^ values range from 0 to 1 and are interpreted just like Pagel's λ [63]. Therefore, *H*^2^ values close to 0 suggest that evolutionary history did not influence effect sizes, whereas values close to 1 suggest that shared ancestry has an influence on effect sizes [61].

We assessed the presence of publication bias [64], which arises when studies reporting significant effects are more likely to be published than those reporting non-significant results, by fitting a modified version of Egger's test [65] that had the residuals of the intercept-only meta-analytical model as the response variable and *V_g_* as the predictor. We considered an intercept that was different from zero as evidence of publication bias in the dataset [61]. As a complement, we used the ‘funnel’ function from the metafor package [56] to visually inspect the relationship between model residuals and effect size precision (i.e. 1/standard error). We also tested for the presence of time-lag bias, which occurs when the magnitude of effect sizes decreases over time as additional studies accumulate and initial estimates are refined [66]. To do so, we fitted a linear regression with *g* as the response variable and the year of publication of each effect size as the predictor variable [61].

Finally, we performed sensitivity analyses to evaluate model fit and robustness. First, we assessed model fit through a visual inspection of the distribution of residuals. Second, we estimated Cook's distance (*D*) with the ‘cooks.distance.rma.mv’ function from the metafor package [56], which computes how removing a certain data point influences a given model. We considered *D* values greater than 1 as extreme [56]. Third, we computed difference in betas (DFBETAS) with the ‘dfbetas.rma.mv’ function from the metafor package [56] to evaluate how the presence or absence of an influential data point influenced model parameters. We considered DFBETAS values greater than 1 as influential cases [67]. When necessary, we refitted models and compared model outputs to determine whether the removal of possibly influential effect sizes changed the qualitative interpretation of our results.

### (c) Bioclimatic data and phylogenetic analyses

Since the relationship between thermal variability and geographical coordinates depends strongly on spatial and temporal scales, and extends beyond the simplistic view that higher latitudes experience greater thermal variation than lower latitudes [36,37], we avoided treating latitude as a proxy for thermal variability. Instead, we extracted site-specific climatic variables associated with thermal variability from Climatologies at High Resolution for the Earth's Land Areas [68] at a spatial resolution of 30 arcsec (approximately 1 km). To maximize biological relevance, we restricted these analyses to studies that sourced animals from nature and provided the geographical coordinates for their collection sites. We used the sp [69] and raster [70] packages to obtain coordinate-specific data on mean diurnal range (BIO2), isothermality (BIO3), temperature seasonality (BIO4) and annual temperature range (BIO7). The mean diurnal range is computed as the average difference between monthly maximum and minimum air temperatures, isothermality quantifies the relative contribution of day–night variation to annual thermal variation, temperature seasonality is given by the s.d. of mean monthly temperatures, and the annual temperature range is a measure of the amplitude between the warmest and coldest months [68,71].

We then assessed correlations among climatic variables to minimize collinearity and identify biologically meaningful predictors of behavioural fever. Mean diurnal range and isothermality emerged as suitable candidates because other thermal variables were strongly correlated with one another (supplementary material, figure S2). For mean diurnal range, higher values pertain to sampling localities characterized by greater day–night temperature fluctuations, whereas isothermality describes the extent to which daily thermal variation contributes to overall annual climatic variability. Values close to 1 pertain to sampling localities with consistent daily temperatures relative to the annual thermal cycle, while values close to 0 indicate larger annual temperature variation compared with the daily range. Thus, these variables capture complementary dimensions of thermal heterogeneity potentially relevant to thermoregulatory decision-making during infection.

To evaluate whether species originating from thermally variable habitats exhibited greater evidence for behavioural fever, we fitted Bayesian phylogenetic generalized linear mixed models (PGLMMs) with the ‘pglmm’ function from the phyr and INLA packages [72,73]. For the global model, we considered the difference between infected and control body temperatures as the response variable and mean diurnal range (scaled and mean-centred), isothermality (scaled and mean-centred) and body mass (log_10_-transformed) as the predictor variables. Because multiple effect sizes were available for some species and sampling effort was uneven across taxa, we included species identity as a random effect and incorporated phylogenetic covariance to account for non-independence among observations and shared evolutionary history. We used a complexity-penalizing prior to automatically determine the best prior distribution for our global model [74]. Because the PGLMM estimates phylogenetic covariance directly, we also calculated Pagel's *λ* [63] to quantify the strength of phylogenetic signal in the model residuals. This differs from the *H*² index estimated in the meta-analysis, which quantifies the proportion of heterogeneity attributable to shared ancestry.

To determine whether climatic variables improved explanatory power, we compared the global model with alternative models containing different combinations of predictors (seven models total). We performed model selection based on the deviance information criterion (DIC), in which higher DIC weight (DIC*_w_*) values indicate better model fit [75]. We assessed the robustness of our best-ranked model through a null-modelling approach that compared whether our observed *R*^2^ value exceeded values expected by chance [76]. We generated 1000 null datasets in which our response variable was randomly permuted while retaining the original predictor variables and phylogenetic structure. We refitted each permuted dataset using PGLMMs and extracted *R*^2^ values to create a null distribution. We compared the empirical *R*^2^ with the null *R*^2^ distribution to assess whether the explanatory power of our best-ranked model exceeded or was similar to that obtained by chance.

## 3. Results

### (a) Dataset

In total, 47 studies passed our inclusion criteria, and we were able to calculate 101 effect sizes for 49 species of amphibians and reptiles (figure 1) [77]. Most of the data we obtained came from reptiles (64 effect sizes for reptiles versus 37 effect sizes for amphibians) with Squamata being the most studied order (89.39% of effect sizes), followed by Testudines (7.58% of effect sizes) and Crocodilia (3.03% of effect sizes); we did not obtain any data from Sphenodontia. In amphibians, Anura was the most studied order (91.89% of effect sizes), followed by Caudata (8.11% of effect sizes); we did not retrieve any data from Gymnophiona. Species origin was balanced between captive-bred (51 effect sizes) and naturally sourced (50 effect sizes) individuals. We obtained exact geographical coordinates for all studies conducted on species sourced from nature. Our final dataset spans six decades of research on behavioural fever (from the 1970s to the 2020s), with most studies being published in the 2010s (supplementary material, figure S3).

**Figure 1.**
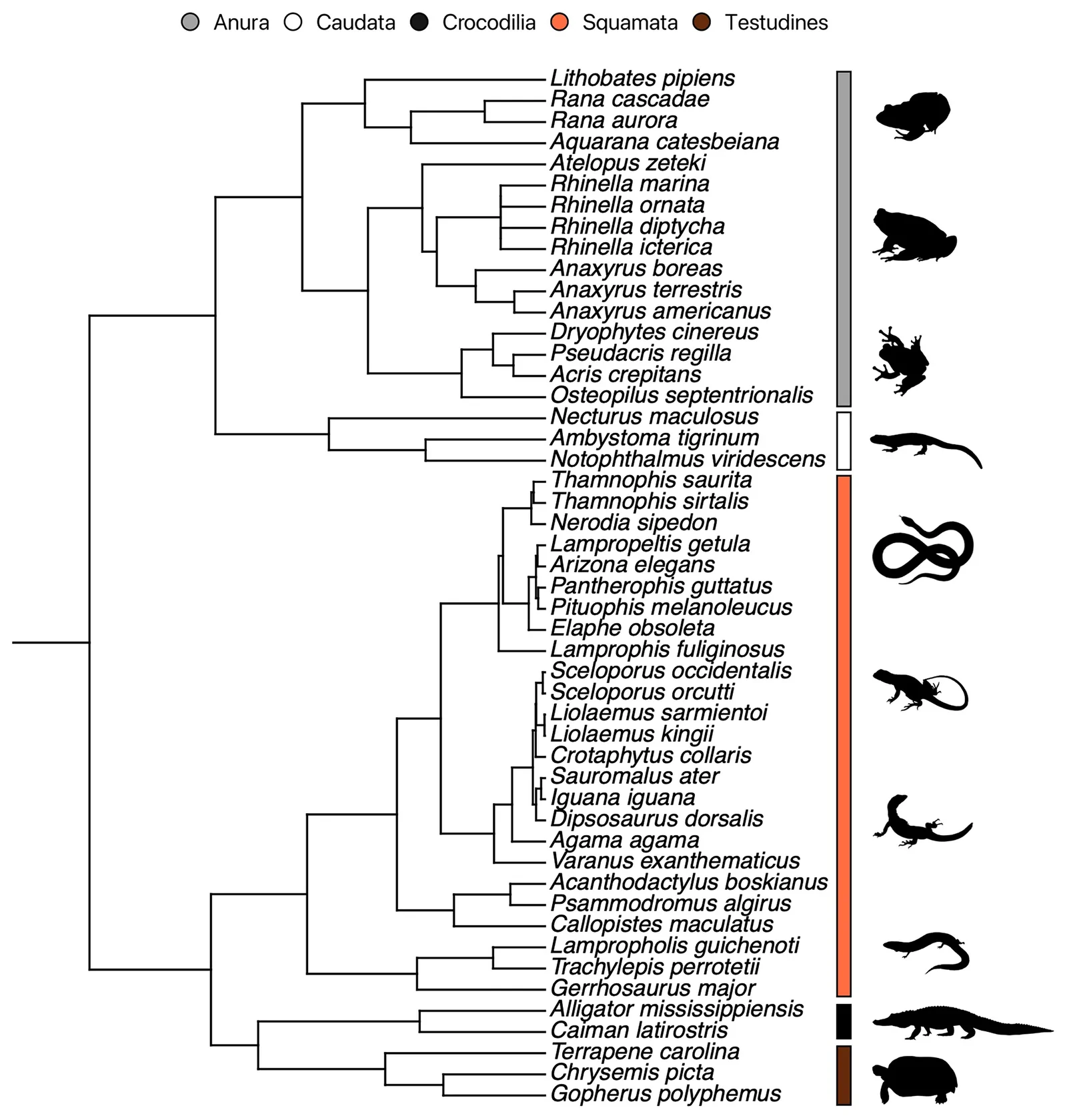
Phylogenetic relationship among the species of amphibians and reptiles represented in our dataset. This tree was created based on two time-calibrated phylogenies of amphibians [58] and reptiles [57]. Amphibian and reptile orders are colour-coded with Anura in grey, Caudata in white, Crocodilia in black, Squamata in orange and Testudines in brown. We obtained silhouettes that represent major amphibian and reptile taxa from PhyloPic's (https://www.phylopic.org/) public repository.

### (b) Behavioural fever differs between amphibians and reptiles and depends on infection type

We present our meta-analytical results as effect size estimates ± standard error (s.e.) [95% CIs]. Based on the intercept-only model, we found that amphibians and reptiles, on average, exhibited behavioural fever when infected (*g* = 0.94 ± 0.40 (0.13, 1.75), *p* = 0.01) (figure 2). We also found that amphibians showed greater evidence of behavioural fever than reptiles (amphibians: *g* = 1.98 ± 0.87 (0.25, 3.70), *p* = 0.02; reptiles: *g* = 0.78 ± 0.80 (–0.81, 2.38), *p* = 0.33) (figure 3a), with no contribution of whether animals were sourced from nature or captivity (*g* = –0.13 ± 0.78 (–1.68, 1.41), *p* = 0.86). Moreover, we detected behavioural fever in animals challenged with bacteria (*g* = 1.22 ± 0.50 (0.23, 2.21), *p* = 0.02), infecting agents in the ‘other’ category (*g* = 1.36 ± 0.65 (0.07, 2.66), *p* = 0.04) and endogenous pyrogens (*g* = 2.54 ± 0.95 (0.66, 4.43), *p* = 0.01) but not viruses (*g* = 0.79 ± 0.82 (–0.83, 2.41), *p* = 0.33) or fungi (*g* = 0.19 ± 0.57 (–0.94, 1.33), *p* = 0.74) (figure 3b). *I*^2^ values from both models were high, suggesting that most of the observed variation remained unexplained by random terms (supplementary material, table S1). There was no evidence of phylogenetic signal in either meta-analytical model (supplementary material, table S1).

**Figure 2.**
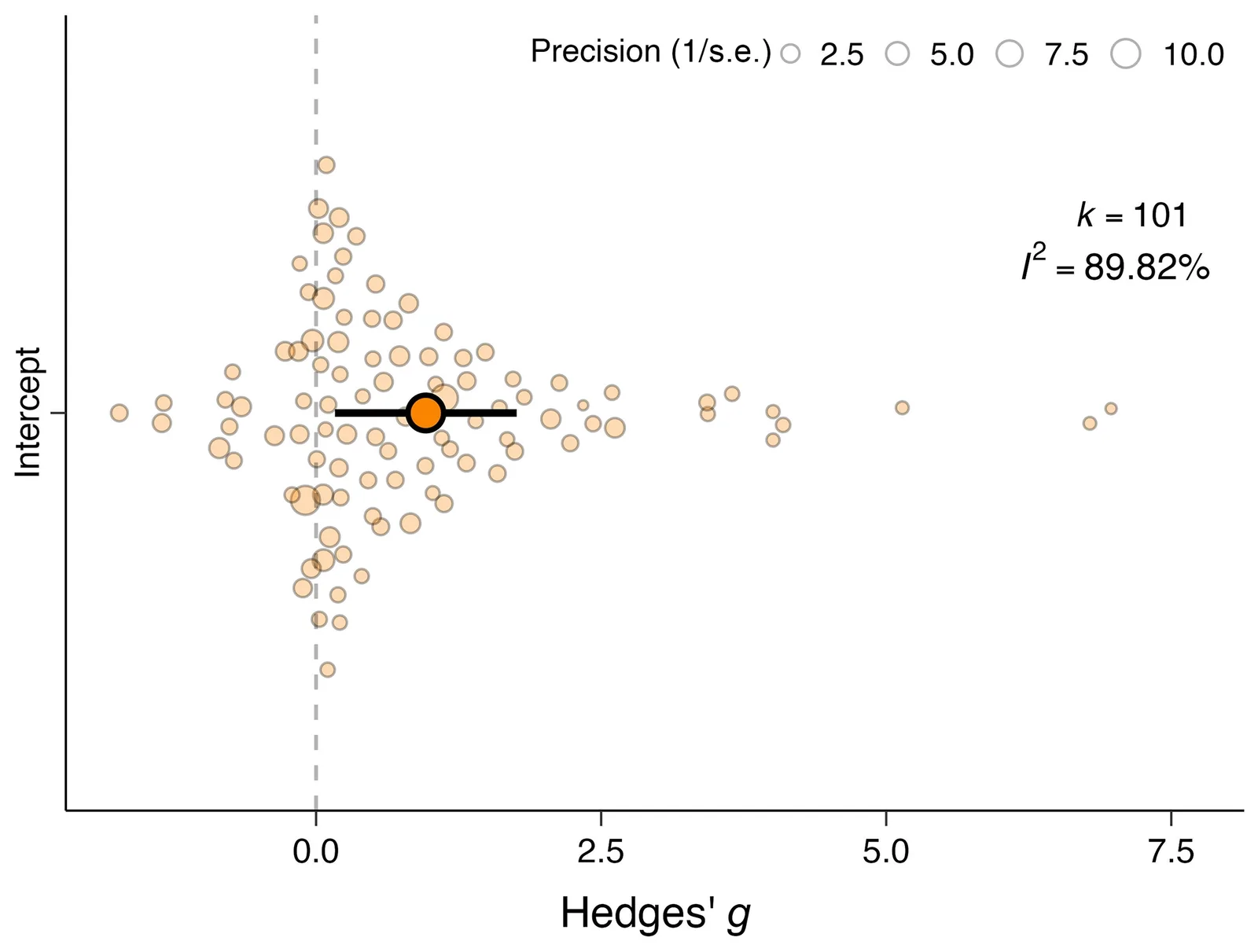
The intercept-only meta-analytical model supports the presence of an overall behavioural fever response in amphibians and reptiles, as evidenced by a positive *g* estimate and 95% CIs that do not overlap the zero. The large orange dot represents the model estimate and the horizontal line depicts the 95% CI. Semi-transparent dots represent individual effect sizes and are vertically jittered around the intercept to avoid overplotting. The vertical dotted lines denote the zero. *k* = number of effect sizes. *I*^2^ = total heterogeneity.

**Figure 3.**
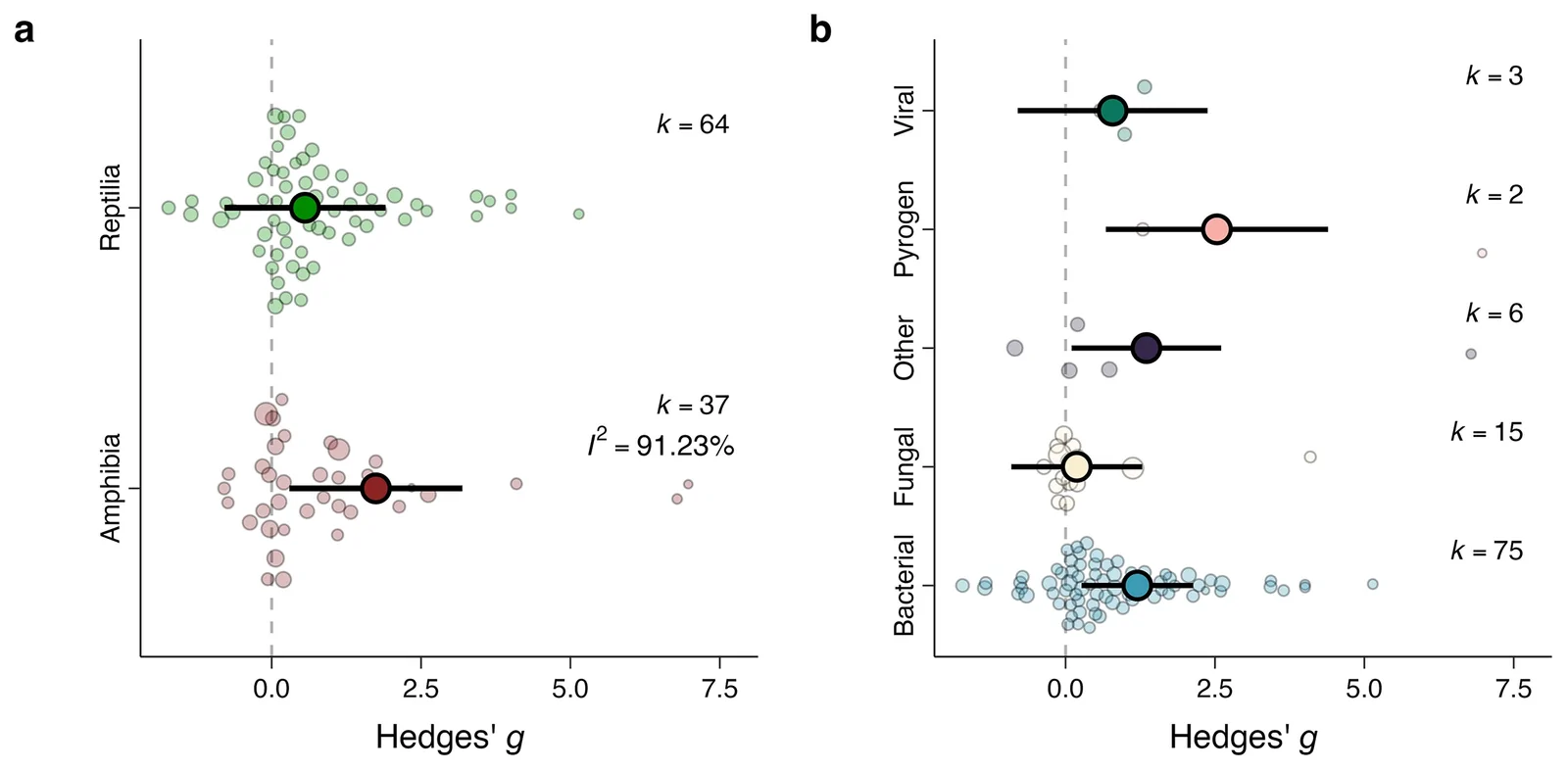
(a) Amphibians display stronger evidence of behavioural fever than reptiles. (b) Infections by bacteria and other agents (nematodes, ticks, fly larvae and heavy metals) are more prone to cause behavioural fever responses than infections by fungi, endogenous pyrogens or viruses. In both panels, large dots represent parameter estimates and the horizontal lines depict the 95% CIs. Semi-transparent dots represent individual effect sizes and are vertically jittered around the intercept to avoid overplotting. The vertical dotted lines denote the zero. *k* = number of effect sizes. *I*^2^ = total heterogeneity.

We found evidence of both publication bias (*β* = –2.18 ± 0.34 (–2.87, –1.48), *p* < 0.001) (supplementary material, figure S4a) and time-lag bias (*β* = 61.41 ± 20.02 (21.68, 100), *p* = 0.002) (supplementary material, figure S4b) in our dataset. Our sensitivity analyses indicated the presence of three possibly influential data points in our meta-analytical models (i.e. DFBETAS > 1). Accordingly, we removed these data from our dataset and refitted our models. Doing so did not alter the qualitative interpretation of our results (supplementary material).

### (c) Daily thermal variation predicts behavioural fever

We report PGLMM results by providing posterior estimates and their corresponding 95% CIs. Species that experienced a wider diurnal thermal range displayed greater shifts in body temperature when infected (posterior estimate = 1.13 (0.37, 1.90), *R*^2^ = 0.68). Because diurnal thermal range was mean-centred and scaled, the posterior estimate indicates that an increase of 1 s.d. in daily thermal variability (approximately 2.50ºC in our dataset) was associated with a 1.13ºC difference in body temperature. In raw units, this translates to an approximate 0.45ºC increase in body temperature for every 1ºC increase in mean diurnal range. This effect was not mediated by neither isothermality (posterior estimate = –0.10 (–1.33, 1.13)) nor body mass (posterior estimate = 0.42 (–0.69, 1.51)) and was associated with an intermediate phylogenetic signal (*λ* = 0.60) (figure 4). Random terms from this model suggested a modest effect for phylogeny (variance = 1.19 (0.34, 8.29)) and residual variation (variance = 3.41 (2.01, 6.53)) with no evidence of residual autocorrelation (Ljung–Box Q Test; Q = 5.62, *p* = 0.77). Our observed *R*^2^ value did not overlap with the randomly generated *R*^2^ distribution (supplementary material, figure S5), supporting the robustness of our model.

**Figure 4.**
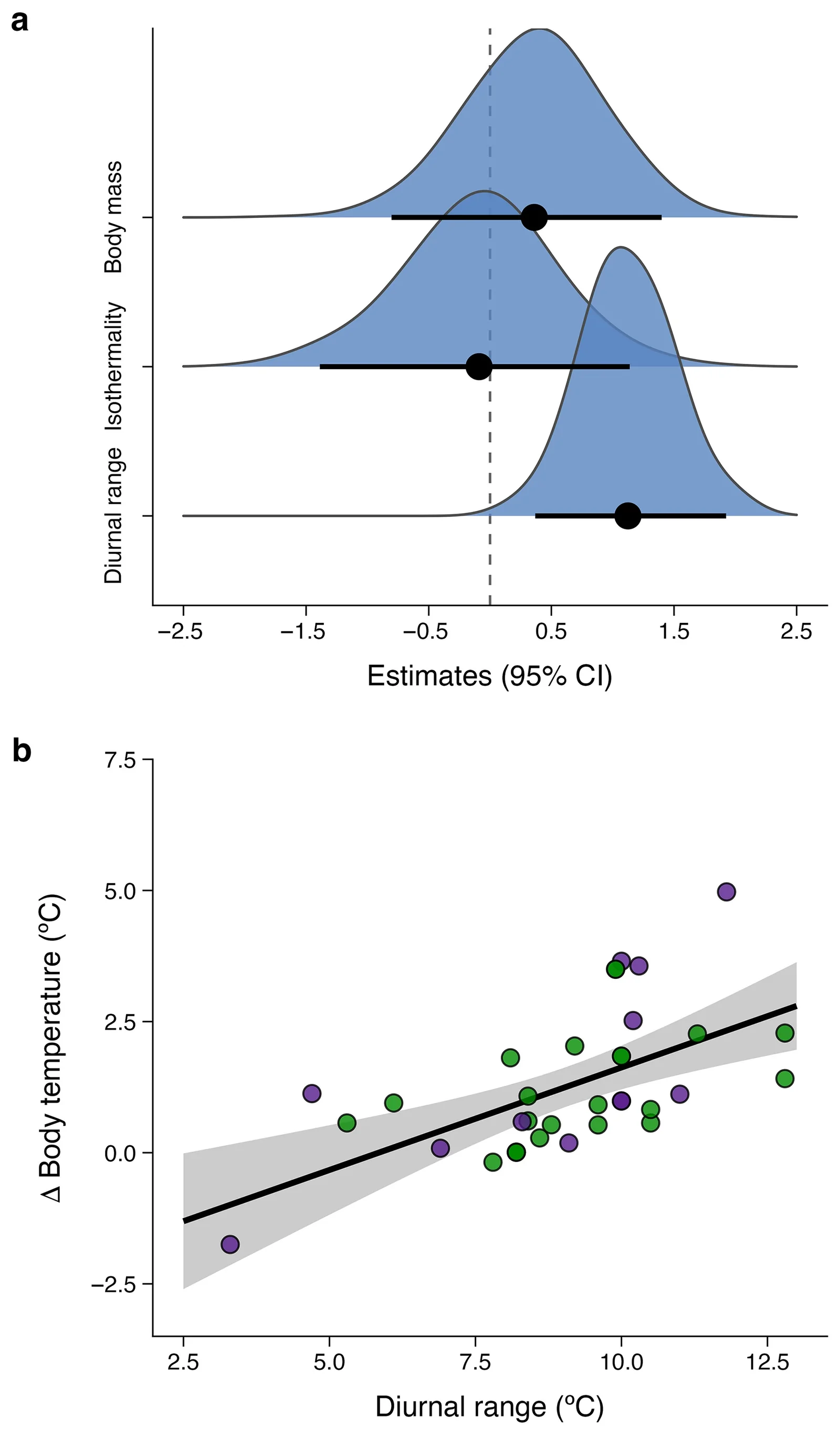
(a) Posterior distributions for fixed terms from the Bayesian phylogenetic generalized linear mixed model evaluating whether species from thermally variable habitats showed greater evidence of behavioural fever. Diurnal range was the only significant model parameter. Black dots represent model estimates and the black horizontal bars represent the 95% CIs. Densities for the posterior distributions are represented in light blue for each model parameter. The vertical dashed line marks the zero. (b) Relationship between shifts in body temperature (Δ body temperature) following an infection and mean diurnal range. Species from habitats that are more thermally variable on a daily basis exhibit greater evidence for behavioural fever than those from more stable habitats (*R*^2^ = 0.68). Dots are colour-coded (purple = reptiles and green = amphibians) and represent individual data points. The black horizontal line shows the predicted relationship between Δ body temperature and mean diurnal range and the grey shaded area represents the 95% CI.

## 4. Discussion

### (a) Behavioural fever is widespread but context-dependent

Our meta-analysis supports the idea that infection is frequently associated with positive thermoregulatory responses across amphibians and reptiles, consistent with behavioural fever as traditionally defined in the literature. This finding is consistent with work demonstrating that amphibians and reptiles can behaviourally elevate body temperature following an immune challenge (reviewed in [10,16,25]). The positive overall effect size across amphibians and reptiles reinforces the view that diverse behavioural responses to temperature constitute a central component of amphibian and reptile biology, including from an immunological standpoint. However, the high heterogeneity observed across models indicates that thermal responses are not uniform. Instead, it suggests that additional aspects, such as ecological and experimental context, must be considered to properly understand the factors that shape fever expression in amphibians and reptiles. Indeed, rather than representing a fixed trait, behavioural fever appears to result from the interaction between conserved immune signalling pathways, the thermal opportunities afforded by the surrounding landscape and the experimental contexts through which thermoregulatory responses are quantified [7,16,24]. In other words, our data suggest that although immune activation may bias thermoregulatory drive toward warmer environmental temperatures via cytokine and prostaglandin signalling pathways [10], the realization of a febrile response, in species that do display this response, should depend largely on the capacity to behaviourally exploit the thermal landscape [32]. This interpretation aligns with conceptual frameworks that emphasize the role of habitat heterogeneity and climatic variability in shaping thermoregulatory responses in tetrapod ectotherms [34,78].

### (b) Why do amphibians show stronger behavioural fever than reptiles?

Contrary to our prediction, we found that amphibians exhibited stronger evidence of behavioural fever than reptiles. A key consideration in interpreting this pattern is the trade-off between thermo- and hydroregulation in amphibians. Because their highly permeable skin and elevated evaporative water loss can increase the hydric costs of warming, amphibians have been traditionally assumed to face stronger hydrothermal trade-offs than reptiles [40,79]. However, studies indicate that some species may possess a greater capacity to balance thermo- and hydroregulation than previously appreciated. Through microhabitat selection and physiological mechanisms that reduce cutaneous evaporative water loss, some species can exploit warm environments while mitigating associated hydric costs [80,81]. In amphibians, behavioural thermoregulation experiments are typically conducted under hydrated and thermally permissive laboratory conditions, often relying on humid thermal gradients that incur low hydrothermal costs [44]. Under such conditions, the hydrothermal trade-offs that exist in nature may be reduced, potentially allowing amphibians to express stronger evidence of heat-seeking behaviours. In turn, this draws attention to a long-standing issue in comparative physiology: what animals can do under experimentally permissive conditions and what they routinely do in nature [82,83]. Accordingly, the stronger behavioural fever observed in amphibians may reflect physiological capacity expressed under permissive laboratory conditions rather than the realized thermoregulatory behaviour observed in the field [but see 82]. Future work would benefit from explicitly comparing laboratory and field body temperatures during infection, while also manipulating hydration state and thermal opportunity to determine how hydrothermal trade-offs constrain the expression of behavioural fever in amphibians.

Differences in baseline thermoregulatory demands between amphibians and reptiles may further contribute to our observed pattern. Indeed, many species of reptiles maintain higher body temperatures and function within narrower temperature ranges than amphibians, particularly among active thermoregulators [41]. Consequently, infected reptiles may already operate near temperatures that optimize immune performance [84], which may reduce the scope for additional warming to be detected as behavioural fever. In such cases, fever may be expressed through subtle adjustments in basking, activity timing or microhabitat use rather than through large increases in mean body temperature. Although these thermoregulatory responses are better documented in reptiles than in amphibians [41,44], the use of pre-post comparisons of body temperature likely does not capture such fine-scale adjustments. Alternatively, amphibians and reptiles can also exhibit regulated decreases in body temperature (i.e. anapyrexia) under certain contexts (e.g. hypoxia), which have been documented as distinct thermoregulatory states alongside fever [10]. While empirical evidence for behavioural anapyrexia following infection is still limited [85], evidence from other contexts suggests that maintaining cooler body temperatures might be an adaptive strategy to conserve energy and mitigate physiological stressors [10,86,87]. In this sense, the absence of an overt fever response in some reptiles may reflect a shift toward cooler body temperatures rather than a lack of positive thermotaxis, underscoring the need for future work to explicitly test whether infection can elicit alternative temperature adjustments or behaviours [25].

A third, non-mutually exclusive explanation for the stronger behavioural fever observed in amphibians compared with reptiles involves methodological differences among studies in terms of how infections are induced, how thermal responses are quantified and the conditions under which such responses are evaluated. Infection protocols vary widely in the literature, encompassing different classes of pathogens (e.g. bacteria, fungi, viruses and parasites), doses, routes of exposure (e.g. injection, immersion, oral inoculation or natural exposure) and timing of temperature measurements relative to infection [25]. Such variation is likely to influence both the magnitude and temporal dynamics of immune activation and consequently whether behavioural fever or other sickness behaviours are expressed [25]. In addition, experimental approaches used to quantify thermal preference or selection differ substantially [44], ranging from linear or circular thermal gradients of varying breadth and resolution to semi-natural enclosures with varying degrees of thermal heterogeneity [88–90]. Although thermal gradient design, spatial complexity and thermal resolution influence the range of temperatures available to animals [44,83], experimental duration may also shape the detectability of fever responses, as studies range from short-term assays lasting only a few hours [91] to multi-day experiments capable of capturing sustained thermoregulatory adjustments [22]. Additional heterogeneity arises from the techniques used to measure body temperature (e.g. cloacal thermometry, implanted data loggers and infrared thermography), which differ in temporal resolution, invasiveness and physiological relevance [92]. A deeper issue, however, concerns whether laboratory-based measurements of behavioural thermoregulation carry the same biological meaning across amphibians and reptiles.

Indeed, behavioural fever, as traditionally defined, is quantified relative to a baseline thermal state [25]. A central question is thus whether the baseline thermal state reflects a strong thermoregulatory preference that is repeatable across experimental settings or whether it is largely contingent on the species under study and the conditions imposed by each experiment [44]. In heliothermic lizards, for example, temperatures selected in laboratory thermal gradients often approximate those maintained in the field [41], such that experimental control temperatures may reflect meaningful thermal set-points. Under these circumstances, the baseline thermal state tends to be robust, repeatable and consistently thermotactic [93]. In many amphibians, however, a comparable baseline thermal state may not exist in the same sense as a stable thermal state, as body temperature can vary continuously according to hydration state, microhabitat conditions, activity level and local thermal opportunity [94,95] or even according to how animals explore the experimental arena [44,83]. Moreover, thermotaxis is not commonly tested in amphibians, meaning that temperatures recorded under laboratory conditions may sometimes reflect exploratory behaviour or avoidance of thermal extremes rather than a stable thermal preference [44,90].

Consequently, whether behavioural fever can be meaningfully quantified depends not only on the magnitude of thermal change after exposure to a pathogen, but also on whether the reference thermal state reflects a pervasive thermoregulatory tendency or a variable thermal behaviour contingent on the experimental context. This distinction suggests that equivalent thermal shifts may not carry the same biological meaning across amphibians and reptiles and that the stronger behavioural fever observed in amphibians could partly reflect greater context dependence in the thermal reference state used to quantify fever. Future work should move beyond simply documenting thermal shifts following infection and explicitly evaluate the biological meaning of the thermal reference states used to infer behavioural fever. This will require validating baseline thermal states through repeated measures and/or control experiments [44,83], field estimates of body temperature and independent tests of thermotactic consistency before laboratory-derived thermal shifts are interpreted as evidence of fever [90]. More broadly, advancing this field will require greater standardization of experimental designs, clearer reporting of infection protocols and thermal environments, and stronger integration between laboratory and field studies to understand when increased temperature functions as a meaningful line of defence against infection.

### (c) Pathogenic and climatic context affect behavioural fever

Our data suggest that the observed variation in the expression of behavioural fever across amphibians and reptiles reflects the influence of both pathogen identity and climatic variability. Pathogens can differ markedly in their thermal sensitivities and the immune pathways they activate, which can influence the magnitude and timing of febrile responses [4]. In our study, behavioural fever was supported following bacterial infections and administration of endogenous pyrogens, whereas fungal and viral challenges elicited weaker and statistically unsupported responses. This finding is consistent with evidence that bacterial challenges and pyrogenic stimuli induce strong pro-inflammatory responses mediated by endogenous and exogenous pyrogens, thereby promoting fever [9]. By contrast, certain fungi may tolerate or even benefit from elevated temperatures [96], while viral infections may cause more variable thermal responses [97]. This pattern is consistent with the thermal tolerance mismatch hypothesis, which posits that the adaptive value of sustaining elevated body temperatures depends on the relative thermal performance curves of host and pathogen [98,99]. In this scenario, febrile responses should be favoured when they cause a disproportionate impairment in pathogen performance relative to host function. However, fever may be absent when pathogens tolerate or benefit from elevated temperatures. It is important to note, however, that bacterial effect sizes were disproportionately represented in our dataset (*k*_bacteria_ = 75, *k*_fungus_ = 15, *k*_other_ = 6, *k*_pyrogen_ = 4, *k*_virus_ = 3), possibly because of the seminal papers that set the stage for the study of behavioural fever in tetrapod ectotherms (e.g. [14,17]). Although pyrogen administration produced comparatively large effect sizes, this category was represented by only two effect sizes and should therefore be interpreted cautiously. As such, although our findings suggest that behavioural fever is not a generic response to infection, it is imperative that future studies test potential fever responses across a broader diversity of pathogens and infection contexts before general conclusions about pathogen-specific thermal strategies can be drawn.

Beyond pathogen identity, our results also demonstrate that climatic thermal variability predicts the expression of behavioural fever after accounting for body size and phylogenetic effects. Species from habitats with greater day–night thermal variation exhibited larger shifts in body temperature following infection, supporting the CVH and comparative work showing that amphibians and non-avian reptiles from thermally heterogeneous habitats exhibit broader thermal tolerances and enhanced behavioural flexibility [27–29]. This suggests that even modest increases in day–night temperature variation can amplify the capacity of amphibians and reptiles to exhibit behavioural fever during infection, potentially enhancing immune performance in species inhabiting thermally variable environments relative to those from thermally stable habitats. We further highlight that this relationship was detected using high-resolution climatic data rather than relying on geographic proxies, reinforcing the idea that biologically relevant measures of thermal variability are essential for properly understanding patterns of thermoregulatory plasticity [30,37]. Nevertheless, our climatic predictors describe broad-scale thermal regimes and do not capture the fine-scale thermal heterogeneity, which can strongly influence body temperature and thermal opportunity [37,39]. Integrating microclimate data with experimental studies of behavioural fever therefore represents an important avenue for future research. In this context, the scale at which thermal variability is quantified is central [39]. From an organismal perspective, diurnal thermal variation directly constrains short-term behavioural decisions and has immediate relevance for fever expression [100], whereas broader seasonal metrics may be less informative for acute immune responses. The relatively weak phylogenetic signal detected in our models supports the idea that the ability to realize behavioural fever is contingent on the prevailing environmental conditions, despite the fact that fever pathways are deeply conserved across the animal tree of life [10,16].

### (d) Biases and limitations

As with any broad-scale study, our conclusions must be interpreted in light of potential sources of bias. We detected evidence of publication bias and time-lag bias [64,66], which is consistent with broader patterns in ecological and evolutionary quantitative syntheses [61]. However, our sensitivity analyses supported the robustness of our main conclusions, as evidenced by the fact that removing potentially influential effect sizes did not alter the qualitative interpretation of our results. Nevertheless, we reiterate that behavioural fever is inherently difficult to quantify across disparate experimental designs, and effect sizes may conflate physiological capacity with behavioural opportunity. Furthermore, our analyses focused on increases in body temperature following infection but did not explicitly account for alternative sickness behaviours, like behavioural anapyrexia, which may be a biologically meaningful defence strategy in certain contexts [10,25]. In this vein, future research should benefit from experimental designs that formally integrate pathogen identity, thermal landscape structure and the temporal dynamics of thermoregulatory responses, thereby allowing fever and alternative sickness behaviours to be evaluated as complementary components of temperature-mediated immune defence.

### (e) Closing remarks

Together, our findings highlight that temperature-mediated defence mechanisms cannot be understood independently of the environments in which they are expressed. The ability to express behavioural fever depends on access to thermally heterogeneous landscapes that allow amphibians and reptiles to elevate body temperature when infected [82], and this reliance makes such defences inherently sensitive to environmental change. Climate change is altering not only mean temperatures but also the magnitude, predictability and spatial structure of thermal variability, often leading to thermal homogenization at local scales [34]. In habitats wherein fine-scale thermal heterogeneity is reduced (e.g. through land-use change and canopy loss), the behavioural capacity to exploit temperature as an immune resource may be hampered even if physiological fever pathways remain intact. Indeed, experimental evidence suggests that infected lizards may die when infected but precluded from behaviourally raising their body temperatures [101]. In a broader context, species that historically relied on behavioural fever may experience increased susceptibility to pathogens if their habitats undergo thermal homogenization in future climatic conditions. Ultimately, our study demonstrates that temperature is not merely a background condition for host–pathogen interactions, but an active and environmentally contingent component of amphibian and reptile immune defence.

## Supplementary Material



## Data Availability

Our data and code can be accessed from [102]. Supplementary material is available online [103].
